# Trends in Stress Throughout Pregnancy and Postpartum Period During the COVID-19 Pandemic: Longitudinal Study Using Ecological Momentary Assessment and Data From the Postpartum Mothers Mobile Study

**DOI:** 10.2196/30422

**Published:** 2021-09-21

**Authors:** Serwaa S Omowale, Andrea Casas, Yu-Hsuan Lai, Sarah A Sanders, Ashley V Hill, Meredith L Wallace, Stephen L Rathbun, Tiffany L Gary-Webb, Lora E Burke, Esa M Davis, Dara D Mendez

**Affiliations:** 1 School of Social Work University of Pittsburgh Pittsburgh, PA United States; 2 Behavioral and Community Health Sciences Graduate School of Public Health University of Pittsburgh Pittsburgh, PA United States; 3 Department of Epidemiology Graduate School of Public Health University of Pittsburgh Pittsburgh, PA United States; 4 Department of Psychiatry University of Pittsburgh Pittsburgh, PA United States; 5 Department of Epidemiology and Biostatistics College of Public Health University of Georgia Athens, GA United States; 6 Department of Health and Community Systems School of Nursing University of Pittsburgh Pittsburgh, PA United States; 7 Division of Internal Medicine Department of Medicine University of Pittsburgh Pittsburgh, PA United States

**Keywords:** COVID-19, ecological momentary assessment, health status disparities, pandemics, postpartum, pregnancy, psychological stress

## Abstract

**Background:**

Stress is associated with adverse birth and postpartum health outcomes. Few studies have longitudinally explored racial differences in maternal stress in a birthing population in the United States during the ongoing COVID-19 pandemic.

**Objective:**

This study aimed to do the following: (1) assess changes in reported stress before, during, and after initial emergency declarations (eg, stay-at-home orders) were in place due to the COVID-19 pandemic, and (2) assess Black-White differences in reported stress in a pregnant and postpartum population from Southwestern Pennsylvania.

**Methods:**

We leveraged data from the ongoing Postpartum Mothers Mobile Study (PMOMS), which surveys participants in real time throughout the pregnancy and postpartum periods via ecological momentary assessment (EMA) and smartphone technology. We analyzed data from a subset of PMOMS participants (n=85) who were either Black or White, and who submitted EMA responses regarding stress between November 1, 2019, and August 31, 2020, the time frame of this study. We divided data into four phases based on significant events during the COVID-19 pandemic: “pre” phase (baseline), “early” phase (first case of COVID-19 reported in United States), “during” phase (stay-at-home orders), and “post” phase (stay-at-home orders eased). We assessed mean stress levels at each phase using linear mixed-effects models and post hoc contrasts based on the models.

**Results:**

Overall mean stress (0=not at all to 4=a lot) during the pre phase was 0.8 for Black and White participants (range for Black participants: 0-3.9; range for White participants: 0-2.8). There was an increase of 0.3 points (*t*_5649_=5.2, *P*<.001) in the during phase as compared with the pre phase, and an increase of 0.2 points (*t*_5649_=3.1, *P*=.002) in the post phase compared with the pre phase (n=85). No difference was found between Black and White participants in the change in mean stress from the pre phase to the during phase (overall change predicted for the regression coefficient=–0.02, *P*=.87). There was a significant difference between Black and White participants in the change in mean stress from the during phase to the post phase (overall change predicted for the regression coefficient=0.4, *P*<.001).

**Conclusions:**

There was an overall increase in mean stress levels in this subset of pregnant and postpartum participants during the same time as the emergency declarations/stay-at-home orders in the United States. Compared to baseline, mean stress levels remained elevated when stay-at-home orders eased. We found no significant difference in the mean stress levels by race. Given that stress is associated with adverse birth outcomes and postpartum health, stress induced by the ongoing COVID-19 pandemic may have adverse implications for birthing populations in the United States.

**International Registered Report Identifier (IRRID):**

RR2-10.2196/13569

## Introduction

### Background

By late 2019, the world had become increasingly aware of the novel coronavirus, SARS-CoV-2, which causes the disease COVID-19. The first case of this coronavirus in the United States was identified in the state of Washington on January 21, 2020 [1]. Shortly thereafter, the US Secretary of Health and Human Services declared a public health emergency on January 31, 2020 [2]. On March 11, 2020, the World Health Organization (WHO) declared COVID-19 a pandemic, signifying the virus had spread to more than one hundred countries [3]. Two days later, the US federal government declared the pandemic a national emergency [2]. On March 16, 2020, the US president and the White House Coronavirus Taskforce members presented guidelines to slow the spread of the virus during a press conference targeted to the US public. These guidelines included listening to local authorities, staying home if sick, and isolating if someone in your household tested positive for the virus [4]. This was federal guidance, but much of the public health intervention needed to address the outbreak was left to local officials. Since there was initially limited federal intervention to address the outbreak, the response to the coronavirus in the United States varied by parish, county, state, and region.

In the initial stages of the pandemic, local government officials had limited tools, knowledge, and resources to address the public’s concern about the virus and to mediate risk to the public. Furthermore, this was a new coronavirus, which impeded the US development and scaling up of diagnostic tests to confirm diagnoses of the virus [5]. Additionally, inconsistent health communication to the public regarding how individuals contracted the virus and which symptoms were indicative of COVID-19 left the public vulnerable to contracting the virus [6]. Since local officials had limited resources to test for the virus and isolate infected individuals, they used other methods to reduce the risk of disease to the public. To slow the spread of the virus and to attempt to avoid overburdening the health care system, some government officials across the nation declared a state of emergency and issued stay-at-home orders as a public health intervention to break the chain of infection by decreasing person-to-person contact. These emergency declarations and policy actions included temporary business closures [7], movement of education to online/remote formats [8,9], and changes in health care system protocols [10]. Employment was affected as well, as 20.4% of US workers were employed in industries impacted by state and local business restrictions aimed at reducing the spread of the virus [11]. In April 2020, employment payrolls fell by 20.5 million people and the unemployment rate was 14.7% [12]. Moreover, uncertainty about the virus and limited mechanisms to mediate the risk to the public greatly disturbed the daily lives of US residents.

Experts have seen a global increase in the incidence of anxiety, depression, posttraumatic stress disorder (PTSD), and psychological distress in the general population during the pandemic [13]. Czeisler et al [14] used representative panel surveys (administered June 24-30, 2020) to assess mental health, substance use, and suicidal ideation among US adults. Of their respondent sample, 40.9% of adults reported at least one adverse mental health condition (symptoms of anxiety or depressive disorder) and 30.9% reported symptoms of trauma or stressors related to the pandemic. Moreover, young adults, Hispanic persons, Black persons, essential workers (eg, health care workers), unpaid caregivers for adults, and persons receiving treatment for pre-existing psychiatric disorders were disproportionately impacted by adverse mental health due to the pandemic [14]. Park et al [15] found common stressors related to COVID-19 included media coverage of viral contagiousness, uncertainty about the length of quarantine or social distancing measures, disruption to social and personal care routines, lack of job security and financial strain, and perceived risk of infection among a sample of US adults. This previous study also found that individuals with caregiver status, younger adults, sexual minorities, and non-White participants were at greater risk for stressors related to the pandemic [15]. Thus, several stressors related to uncertainty about the pandemic impacted populations in the United States, which was felt disproportionately among marginalized communities (ie, Black people, LGBTQ people) and individuals experiencing adverse mental health prior to the pandemic.

Given that psychological stress is associated with adverse birth outcomes and maternal health, stress induced by the COVID-19 pandemic may have implications for perinatal and birthing populations in the United States. Several international studies have reported an increase in psychological stress and psychiatric symptoms during the COVID-19 pandemic among childbearing populations; however, few studies are based in the United States [16-18]. Preis et al [19] found that pregnant people in the United States (recruited late April 2020) reported mild (35.6%), moderate (21.6%), and severe (21.7%) anxiety. Using a pandemic-related stress scale (Pandemic-Related Pregnancy Stress Scale), they found that pandemic preparedness stress (OR 1.75, 95% CI 1.35-2.26) and anxiety related to perinatal COVID-19 infection stress (OR 1.55, 95% CI 1.28-1.88) were associated with a greater likelihood of moderate or severe anxiety symptoms after adjustment for sociodemographic, medical, and obstetrical variables. In a follow-up study (conducted April-May 2020), pandemic preparedness stress (30%) and perinatal infection stress (27.2%) were associated with income loss, prenatal care disruption, and perceived COVID-19 infection among pregnant people [20]. A mixed methods study conducted March to April of 2020 found that 60% of surveyed pregnant participants (n=27) reported experiencing moderate or severe anxiety symptoms and 68% reported moderate stress. In the qualitative results (N=31), participants reported uncertainty related to prenatal care, stress related to the risk of COVID-19 infection, disruption of birth plans, and lack of postpartum support [21]. One study also found that over 50% of pregnant participants reported increased stress related to food insecurity, loss of job or household income, and loss of childcare [22]. Another study found COVID-19 health worries (eg, fear of infection) and grief (eg, loss of meaningful experiences) were associated with clinically significant levels of depression, generalized anxiety, and PTSD among pregnant and postpartum participants. This study also found that participants who reported pre-existing mental illness diagnoses were more likely to report these symptoms [23]. These studies suggest that stress related to lack of support, income loss, uncertainty regarding prenatal/postnatal care, perceived risk of COVID-19 infection, and inability to meet basic needs (eg, secure food) were associated with reports of increased stress and symptoms of mental health illnesses among perinatal populations in the United States. Prior studies based in the United States have not examined longitudinal changes in stress among perinatal populations by race, thus creating the impetus to examine these experiences in this population.

### Objective of Study

This study is a secondary analysis of data from the Postpartum Mothers Mobile Study (PMOMS), a prospective longitudinal study examining factors associated with racial disparities in postpartum weight retention and cardiometabolic health. PMOMS uses smartphone technology to remotely collect survey data via ecological momentary assessment (EMA). EMA allows for the assessment of study participants’ experiences, moods, and behaviors in the context of their natural environment and in real time [24]. The EMA component of PMOMS enabled the continuation of primary data collection during the COVID-19 pandemic, since participants answered survey questions via smartphone. Further details about the study are published elsewhere [25-27].

We investigated changes in reported stress during different phases of the pandemic in a sample of US pregnant and postpartum people. The aims of this manuscript are the following: (1) assess changes in reported stress before, during, and after initial emergency declarations/stay-at-home orders were in place, and (2) assess whether reported stress differed by race during these time periods over the COVID-19 pandemic. We hypothesized the following: (1) all participants would report higher mean stress levels in the during phase compared to the pre phase; (2) in the post phase, reported mean stress levels would return to pre phase levels for all participants; and (3) reported mean stress levels would increase for all participants from the pre phase to the during phase, but the change in reported stress levels would be higher for Black participants than for White participants.

## Methods

### Study Design

PMOMS is an ancillary study to GDM^2^ (Comparison of Two Screening Strategies for Gestational Diabetes) [28-30], a randomized controlled trial conducted in a single women’s hospital in Southwestern Pennsylvania. In addition to the participants recruited to PMOMS from the GDM^2^ clinical trial (n=284), participants were also directly recruited into the PMOMS study (n=29). The study participants are recruited during the second and third trimester (18-28 weeks of gestation) and followed up to 1 year postpartum. Once participants consented to the study, they completed baseline surveys, received smartphones and a smart scale, and downloaded a companion app to weigh themselves. Participants completed EMA surveys for the duration of the study. The protocol for PMOMS was approved by the Human Research Protection Office at the University of Pittsburgh.

### Setting and Participants

**Figure 1:**
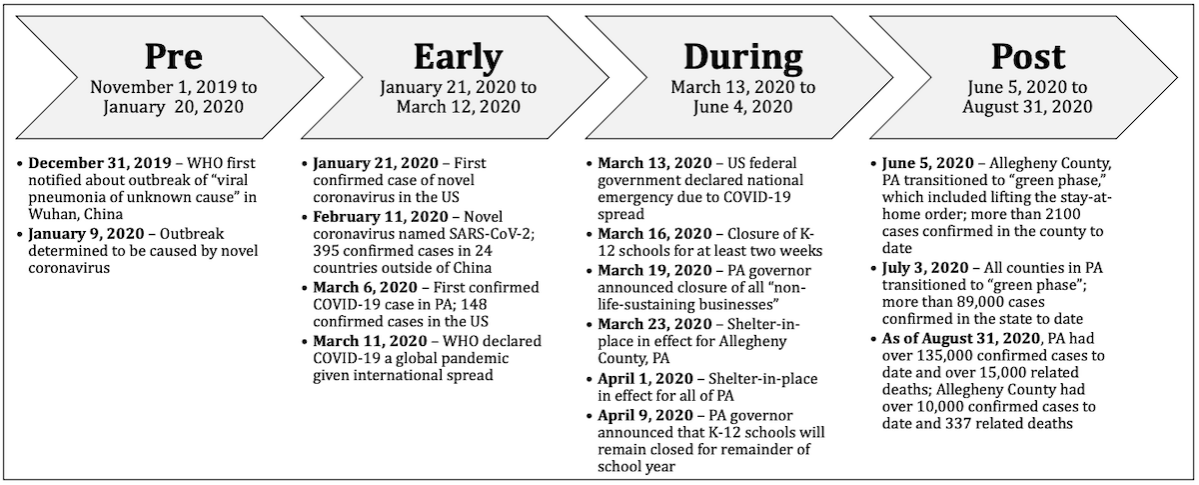
Phases of COVID-19 response as applied in the present analysis, based on administrative actions at the national, state, and county levels. Data obtained from [32-34]. PA: Pennsylvania; WHO: World Health Organization.

A subset of participants from PMOMS contributing data from November 1, 2019-August 31, 2020, served as the analytic sample (n=85). We divided the study period into four phases based on significant events in the COVID-19 pandemic timeline in the United States, Pennsylvania, and Allegheny County. Most of the study participants (n=81) lived in Allegheny County during the study period. The “pre” phase (November 1, 2019-January 20, 2020; 81 days) represents a reference baseline period before the first confirmed case of COVID-19 in the United States. The “early” phase (January 21, 2020-March 12, 2020; 52 days) began on the day the first COVID-19 case was reported in the United States [1]. The “during” phase (March 13, 2020-June 4, 2020; 84 days) began on the day COVID-19 was declared a national emergency and Pennsylvania officials implemented statewide stay-at-home orders (closure of all businesses that were not life sustaining) [31]. This period also included remote and online educational learning for public schools, business closures, and the introduction of stay-at-home orders specifically in Allegheny and surrounding counties [32]. The “post” phase of this study (June 5, 2020-August 31, 2020; 88 days) covered the transition from previous stay-at-home orders to the restricted opening of nonessential businesses (eg, bars, gyms) in Allegheny County (Figure 1).

### Measurements

#### Overview

Participants used a smartphone to complete daily EMA surveys via a web-based app. EMA data were collected at the beginning of day, end of day, and random times throughout the day. Participants chose the timing of the beginning of day and end of day surveys. There were at least nine hours between these two surveys. Using a random sampling design (described in detail elsewhere [25]), random EMA surveys were delivered 0-3 times per day between the beginning of day and end of day survey times, targeting a mean of one random assessment per day over a 7-day period. Data collected via the GDM^2^ study included baseline demographic information.

#### Primary Outcome

The primary outcome of interest in this study was mean self-reported stress levels. Self-reported stress levels were assessed in random EMA surveys using a single item from the Cohen Perceived Stress Scale [35]. The item was adapted, as participants were asked to rate their current level (“right now” as opposed to “in the last month”) of nervousness or stress. In each random survey, participants were asked to “rate if you are feeling nervous or stressed right now.” The response scale ranged from 0 (not at all) to 4 (a lot).

#### Main Predictors

The main predictors in the model were stage (whether the participant was in the pregnancy or postpartum stage), race, and phase of the COVID-19 pandemic timeline (ie, study phases as described in the Setting and Participants section). Participants self-reported race via self-administered electronic surveys at the baseline visit (18-28 weeks of gestation).

#### Covariates

We selected covariates based on the literature and our understanding of stress during pregnancy [36,37]. Education level, employment status, annual household income, marital status, and maternal age were included as covariates. All covariates except maternal age were dichotomized, with education dichotomized as less than college versus college degree or higher, employment status as unemployed versus employed, annual household income as earning less than $50,000 per year versus over $50,000 per year, and marital status as unmarried versus married.

### Analytical Sample

The overall PMOMS population included 313 participants. Of these, 197 (62.9%) completed PMOMS prior to the time frame of this study (November 1, 2019-August 31, 2020), leaving a total of 116 participants (37.1%). Of those, 24 (20.7%) did not complete EMA surveys with stress data, resulting in 92 participants (79.3%) with stress data. We excluded participants that identified as Asian (n=5) or more than one race (n=2) since our primary aim was to examine Black-White differences in stress levels during the study period; this resulted in a total of 85 participants in the final analytic sample. The analytical sample included participants that self-identified as White (n=53, 62%), Black or African American (n=31, 37%), and African (n=1, 1%) who completed EMA surveys. The participants included in the analytic sample were more likely to be White, be married, be college educated, be employed full-time or part-time, and have a household income of $50,000 or higher compared to participants that were excluded.

### Statistical Methods

Participants were included in the analytic sample for a phase if they contributed at least one survey during that phase. Not all participants contributed surveys in every phase. Descriptive analyses reported frequencies and percentages of categorical demographic variables including race, ethnicity, student status, education level, employment status, annual household income, and marital status, and mean (SD) for the continuous variable maternal age. We also report mean numbers of random survey responses contributed per participant in each of the four phases (overall and by race) and unadjusted mean stress levels in each of the four phases (overall and by race). Variance inflation factors (VIF) were used to assess the multicollinearity among education level, annual household income, employment status, and marital status. Since all VIFs were less than 5, we included all covariates in the model.

We used linear mixed-effects models with random subject effects to describe the effect of COVID-19–related emergency declarations/stay-at-home orders on repeated measures of stress levels in random EMA assessments. The initial adjusted model included stage, race, study phase, and the interaction between race and study phase. The fully adjusted model included stage, race, study phase, the interaction between race and study phase, education level, employment status, household income, marital status, and maternal age. *F* tests were used to determine the significance of fixed effects. Mean stress levels by phase and race were computed for each model. Post hoc contrasts based on the models were used to address the study aims. Additionally, since the stress variable was based on a scale of 0 to 4, we conducted analyses using an ordinal generalized linear mixed-effects model with random subject effects (see Multimedia Appendix 1). Due to small numbers of responses in stress levels 3 and 4, we collapsed the original 5 levels into 3 categories, where stress level 0=0, 1=1 and 2, and 2=3 and 4. This model included the same variables as in the adjusted linear mixed-effects model. We conducted all analyses using SAS (version 9.4; SAS Institute Inc).

## Results

Table 1 presents the baseline demographic characteristics of the study participants who contributed EMA data in each phase. As participants completed the PMOMS study, the sample size declined across the COVID-19 pandemic phases. One Black participant withdrew from the study but contributed survey data to the pre phase prior to doing so. The study sample had a mean age of 29.9 (SD 4.8) years and was mostly White (n=53, 62%), married (n=48, 57%), and employed full-time or part-time (n=63, 74%), with an income greater than $50,000 (n=43, 51%) and a college degree or higher (n=44, 52%). The percentage of Black participants contributing to the data in each phase ranged between 34% (n=12, during phase) and 42% (n=10, post phase), whereas the percentage of White participants contributing data ranged between 58% (n=14, post phase) and 66% (n=23, during phase).

Table 2 displays the mean number of EMA surveys contributed by participants overall and in each phase, stratified by race. White participants contributed a higher mean number of surveys overall (overall mean 63.7) and in each phase (mean range 25.2-57.9) compared to Black participants (overall mean 43.9; mean range 15.4-26.1).

**Table 1:** Frequencies of participants contributing data in each phase by demographic variable and stratified by race (Black and White)^a^.

Variable	All (N=85)		Pre phase (n=66)			Early phase (n=52)			During phase (n=35)			Post phase (n=24)	
	Black (n=32; 38%)	White (n=53; 62%)	Black (n=23; 35%)	White (n=43; 65%)	Black (n=19; 37%)		White (n=33; 64%)	Black (n=12; 34%)		White (n=23; 66%)	Black (n=10; 42%)		White (n=14; 58%)
Hispanic, n (%)	0 (0)	3 (4)	0 (0)	3 (5)	0 (0)		1 (2)	0 (0)		1 (3)	0 (0)		0 (0)
Current student, n (%)	3 (4)	4 (5)	3 (5)	4 (6)	1 (2)		2 (4)	1 (3)		1 (3)	0 (0)		0 (0)
College degree or higher, n (%)	6 (7)	38 (45)	5 (8)	31 (47)	2 (4)		26 (50)	2 (6)		18 (51)	1 (4)		11 (46)
Currently employed, n (%)	18 (21)	45 (53)	17 (26)	35 (53)	9 (17)		29 (56)	3 (9)		19 (54)	1 (4)		13 (54)
Annual household income >$50,000, n (%)	3 (4)	40 (47)	3 (5)	32 (49)	1 (2)		26 (50)	1 (3)		18 (51)	0 (0)		11 (46)
Married, n (%)	6 (7)	42 (50)	5 (8)	33 (50)	2 (4)		29 (56)	2 (6)		20 (58)	1 (5)		12 (50)
Maternal age (years), mean (SD)	28.2 (4.8)	30.9 (4.5)	28.7 (5.0)	30.9 (4.3)	28.8 (3.9)		32.0 (4.6)	27.7 (3.5)		32.4 (5.2)	27.7 (3.5)		32.4 (5.2)

^a^All covariates were measured at baseline. Black was defined as Black/African American or African. Percentages (in parentheses) were calculated for all participants contributing data in each phase.

**Table 2:** Distribution of number of completed surveys per participant by phase and race (N=85).

Phase and race		Median	Minimum	Maximum
**All (N=85)**				
	Black (n=32)	33	1	153
	White (n=53)	55	1	199
**Pre (81 days; n=66)**				
	Black (n=23)	25	1	74
	White (n=43)	38	2	75
**Early (52 days; n=52)**				
	Black (n=19)	13	1	34
	White (n=33)	23	3	49
**During (84 days; n=35)**				
	Black (n=12)	18.5	1	59
	White (n=23)	58	2	73
**Post (88 days; n=24)**				
	Black (n=10)	20	3	61
	White (n=14)	62.5	1	87

The distribution of unadjusted mean stress levels for Black and White participants is shown in Table 3. The overall mean stress levels between Black and White participants were not different during the pre phase; however, differences were observed at each of the other phases.

**Figure 2:**
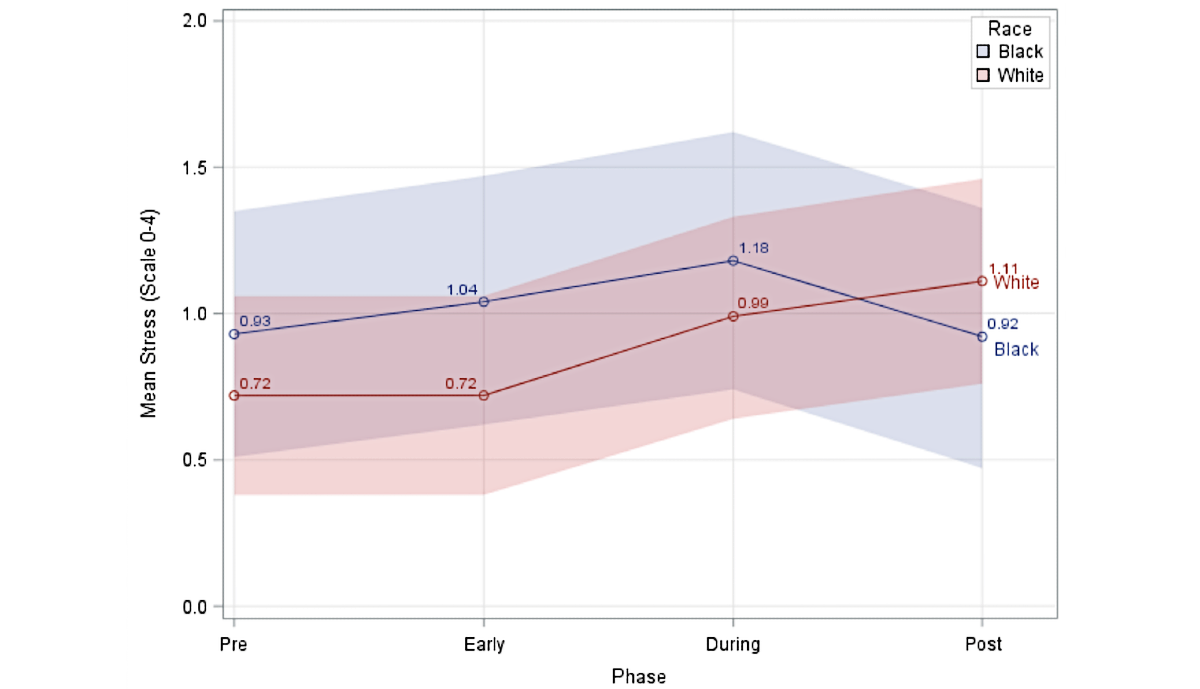
Mean level of stress for each phase by race, adjusted for marriage, education level, employment, and income level.

Since results from the unadjusted model were very similar to those from the adjusted model, results from the adjusted model were used to address the three main hypotheses of the study. Based on the adjusted model, the interaction term regarding race and study phase was statistically significant (*F*_1,3_=14.8, *P*<.001). Figure 2 shows the mean stress levels for Black and White participants.

**Table 3:** Distribution of unadjusted mean stress levels by phase and race (N=85).

Phase and race		Mean	Minimum	Maximum
**Pre (n=66)**				
	Black (n=23)	0.8	0	3.9
	White (n=43)	0.8	0	2.8
**Early (n=52)**				
	Black (n=19)	0.8	0	2.9
	White (n=33)	0.9	0	2.7
**During (n=35)**				
	Black (n=12)	1.4	0	4.0
	White (n=23)	1.1	0	4.0
**Post (n=24)**				
	Black (n=10)	0.5	0	1.8
	White (n=14)	1.3	0.1	4.0

Post hoc contrasts regarding all participants, based on the linear mixed-effects model, showed an increase in mean stress level of 0.26 points (*t*_5649_=5.2, *P*<.001) in the during phase compared to the pre phase, and an increase of 0.2 points (*t*_5649_=3.1, *P*=.002) in the post phase compared to the pre phase (Table 4). Results showed that there was no difference between Black and White participants in the change in mean stress from the pre phase to the during phase (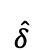
=–0.02, *P*=.90). Given the significant race and phase interaction and the mean stress trajectories in Figure 2, which showed that mean stress increased between the during and post phases for White participants and decreased for Black participants between these phases, we conducted an ancillary analysis focused on a final contrast between Black and White participants regarding the change in reported stress levels between the during and post phases. There was a significant difference between Black and White participants in the change in mean stress levels from the during phase to the post phase (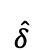
=0.4, *P*<.001).

In the additional analyses conducted using ordinal generalized linear mixed-effects models, the results were consistent with those from the linear mixed-effects model for the first and third hypotheses. However, the ordinal generalized linear mixed-effects model supported the second hypothesis, while the linear mixed-effects model did not. Further details regarding the results from the ordinal generalized linear mixed-effects model can be found in Multimedia Appendix 1.

**Table 4:** Phase-to-phase comparison of mean stress in the adjusted model.

Phase comparison	Difference in adjusted mean stress (SE)	*T* value (*df*)	*P* value
During to pre	0.3 (0.1)	5.2 (5649)	<.001
Post to pre	0.2 (0.1)	3.1 (5649)	.002

## Discussion

### Principal Results

We found an increase in mean reported perceived stress levels during initial emergency declarations and stay-at-home orders in Allegheny County, Pennsylvania, compared to baseline (pre phase) among study participants. This finding supports our first hypothesis that participants would report increased stress levels during the period of initial emergency declarations/stay-at-home orders (during phase), which were enacted to decrease the public’s risk of infection with COVID-19 due to limited knowledge and resources to address the outbreak. The second hypothesis predicted that reported stress levels would return to baseline (pre phase) in the post phase (when stay-at-home orders eased), as residents could resume some of their normal daily activities. However, we found that stress levels remained elevated during this period and did not return to baseline. Our third hypothesis anticipated that Black participants would report higher stress levels from the pre phase to the during phase than White participants, based on previous studies indicating that Black people reported higher stress levels during pregnancy than non-Black people [38,39]. We found no significant difference in mean stress level between Black and White participants during the phases (pre, early, and during) of the study. We also found that only Black participants’ mean levels of stress were near baseline levels during the post phase. In fact, mean stress levels among White participants increased in each phase of the study (pre, early, during, and post), and did not return to baseline levels. In our ancillary analysis (Multimedia Appendix 1), we found a significant Black-White difference in mean stress levels from the during to the post phase, when mean stress levels decreased for Black but not White participants.

The psychological, economic, and other short-term and long-term consequences of the COVID-19 pandemic are still emerging. During our study period, the US public experienced varying risk levels based on community spread of the virus. Uncertainty related to anticipated surges of cases without a vaccine or treatment to sufficiently reduce risk of illness or death was a potential stressor. Additionally, unemployment—even briefly—may have longer-term financial consequences as families recover from income loss, including the risk of housing insecurity. As noted during the COVID-19 pandemic in the United States, even as restrictions on nonessential businesses eased, the easing of restrictions was not a panacea. The unemployment rate was 8.4% when public health–related restrictions eased in August 2020, which was still 4.9 percentage points higher than it was in February 2020, prior to stay-at-home orders [40]. Changes in employment and formal and informal resources during the different phases of the pandemic may have contributed to the increasing reported stress levels for Black and White participants. We collected limited sociodemographic data longitudinally, which prevented us from elucidating differences in reported stress levels for both Black and White participants based on changes in sociodemographic characteristics (eg, employment). In addition, there may be racial differences in completion rates of surveys. However, we do not know if survey completion rates were an effect of the pandemic or a true racial difference. Although this is a limitation, our study findings provide important insight into the experiences of pregnant and postpartum people during the pandemic.

Previously, two studies found that working from home, spending more time with a new baby, saving money, managing expectations, having access to outdoor spaces, and practicing healthy behaviors were all positive coping mechanisms among US pregnant and postpartum people during the pandemic [20,21]. However, parents who continued to work during stay-at-home orders, returned to work, or telecommuted as a work option potentially experienced disruptions or challenges to childcare due to uncertainty regarding the reopening of schools, remote learning for children, and disruptions in other childcare options [9,41]. These factors as well as loss of household income, difficulty meeting basic needs (eg, rent/mortgage), and other unmet needs (eg, food insecurity) may contribute to experiences of stress. Additionally, national protests against racism and calls for police reform in the wake of the killing of George Floyd ensued over the summer of 2020. The inability to cope with unmet needs related to the pandemic in tandem with the effects of state-sanctioned violence had the potential to exacerbate experiences of stress among childbearing people. Further research is necessary to establish whether resources to cope with these challenges contributed to racial differences in reported stress levels in perinatal populations and to identify the protective factors that were most beneficial to different racial groups.

It is important to note that we modeled stress both as an ordinal and a continuous variable. The results from these two models were consistent for the first and third hypotheses. The discrepancy between results for the second hypothesis may be attributed to the difference in how stress levels were categorized. In the ordinal generalized mixed-effects model, response categories were collapsed into 3 categories from the original 5 levels due to smaller numbers of responses in the higher stress categories (stress levels 3 and 4). Thus, the ordinal generalized mixed-effects model could not detect the nuanced changes from one stress level to the next given the collapsed categories.

### Limitations

We are aware that our study findings have some limitations. First, this study did not collect data on changes in sociodemographic information over time, but baseline measures provided some indication of participants’ status. As previously stated, this was a limitation in examining racial differences in our sample. Second, our overall sample size decreased from one phase of the study to the next, especially for Black participants. In addition, Black participants contributed fewer surveys throughout the study period, and their responses were the lowest in the post phase. Our findings indicated that Black participants’ mean stress levels returned to baseline during the post phase. Lower survey responses among Black participants throughout the study period could bias findings, especially if the reduction in response rates was due to experiences of stress during the study period. Finally, since our sample was drawn from one county served by one maternity hospital in Pennsylvania, our findings may not be generalizable to other settings.

### Strengths

Several strengths of this study should be noted. The participants were already enrolled in an ongoing longitudinal study, which allowed for the examination of stress over significant phases of the COVID-19 pandemic in the United States, particularly in a diverse population in southwestern Pennsylvania. The use of EMA methods and smartphone technology meant that survey responses were obtained in real time and in the social context of the participants. Additionally, we used a random sampling design in administering random assessments to provide a representative sample of participants' survey responses over the study interval. Moreover, survey responses collected in real time via smartphone technology provided insight into participants’ experiences of stress during the initial emergency declarations related to the pandemic and stay-at-home orders intended to reduce the public’s risk of infection.

### Conclusions

In this paper, we explored racial differences in stress over time among childbearing people during the COVID-19 pandemic using EMA data collection methods via smartphone technology. To our knowledge, this is the first study using these methods to examine stress over time in a diverse US sample of childbearing people. Evidence from this study suggests there are racial differences in experiences of stress during the pandemic. Moreover, differences in socioeconomic status and support systems, such as marriage, may influence the degree of the impact of the COVID-19 pandemic. The impact of COVID-19 on US residents is ongoing and the risk of infection is still a public concern. Our research highlights the need for medical and public health practitioners to understand stress among perinatal populations during an ongoing pandemic and public health emergency, so they know how to intervene to reduce adverse maternal health outcomes. Ongoing research is needed to understand the enduring and long-term impact of the COVID-19 pandemic on childbearing individuals in the United States, and how best to address these concerns for different populations.

## Appendix

Multimedia Appendix 1 Ordinal generalized linear mixed-effects modeling.

## Abbreviations

EMA: ecological momentary assessment
GDM^2^: Comparison of Two Screening Strategies for Gestational Diabetes Trial
PMOMS: Postpartum Mothers Mobile Study
PTSD: posttraumatic stress disorder
VIF: variance inflation factor
WHO: World Health Organization
