# Supplementary material for: Trends in Stress Throughout Pregnancy and Postpartum Period During the COVID-19 Pandemic: Longitudinal Study Using Ecological Momentary Assessment and Data From the Postpartum Mothers Mobile Study
Source: JMIR Ment Health. 2021 Sep 21;8(9):e30422. doi: 10.2196/30422 (PMC8457341; doi:10.2196/30422)
Supplement: Multimedia Appendix 1 [file mental_v8i9e30422_app1.docx]

# APPENDIX 1 – Ordinal generalized linear mixed-effects modeling

Since the stress variable was based on a scale of 0 to 4, we also conducted analyses using ordinal generalized linear mixed-effects modeling. Due to the small numbers of responses in stress levels 3 and 4 (Table A), we collapsed the five levels into three as shown in Table B. We collapsed the five levels into three, where stress level 0=0, 1=1 and 2, and 2=3 and 4. The ordinal generalized linear mixed-effects model included stage, race, study phase, the interaction between race and study phase, education level, employment status, household income, marital status, and maternal age.

Table A. Frequency and percentage of self-reported stress responses in each COVID-19 phase (linear mixed model).

| **Stress Scale Response** | **Pre-Phase**  **n (%)** | **Early-Phase**  **n (%)** | **During-Phase**  **n (%)** | **Post-Phase**  **n (%)** |
| --- | --- | --- | --- | --- |
| **0: not at all** |  |  |  |  |
|  | 1344 (60.3) | 691 (59.6) | 827 (53.9) | 649 (57.9) |
| **1** |  |  |  |  |
|  | 490 (22.0) | 236 (20.4) | 269 (17.5) | 215 (19.2) |
| **2** |  |  |  |  |
|  | 238 (10.7) | 145 (12.5) | 246 (16.0) | 117 (10.4) |
| **3** |  |  |  |  |
|  | 97 (4.4) | 63 (5.4) | 106 (6.9) | 100 (8.9) |
| **4: a lot** |  |  |  |  |
|  | 59 (2.7) | 24 (2.1) | 86 (5.6) | 40 (3.6) |

Table B. Frequency and percentage of self-reported stress responses in each COVID-19 phase (ordinal generalized linear mixed model).

| **Stress Scale Response** | **Pre-Phase**  **n (%)** | **Early-Phase**  **n (%)** | **During-Phase**  **n (%)** | **Post-Phase**  **n (%)** |
| --- | --- | --- | --- | --- |
| **0** |  |  |  |  |
|  | 1344 (60.3) | 691 (59.6) | 827 (53.9) | 649 (57.9) |
| **1** |  |  |  |  |
|  | 728 (32.7) | 381 (32.9) | 515 (33.6) | 332 (29.6) |
| **2** |  |  |  |  |
|  | 156 (7.0) | 87 (7.5) | 192 (12.5) | 140 (12.5) |

Participants in the during-phase had 2.3 times higher odds of being in a one-higher stress category compared with participants in the pre-phase (OR=2.3, 95% CI: 1.5, 3.3). This finding supported our first hypothesis that participants would report increased stress levels during the period of initial emergency declarations/stay-at-home orders (during-phase) as compared to the pre-phase. This finding was consistent with the results from the linear mixed-effects model, where participants showed an increase in mean stress level of 0.3 points (t=5.2, d.f.=5649, *P*<.0001) in the during-phase as compared with the pre-phase.

Regarding the second hypothesis—that reported stress levels would return to baseline (pre-phase) in the post-phase—the model indicated no difference in stress levels between the pre- and post-phases (OR=1.3, d.f.=5648, 95% CI: 0.8, 2.2). Although this outcome supported our second hypothesis, it was different from the results from the linear mixed-effects model, which showed that participants reported an increase in mean stress level of 0.2 points (t=3.1, d.f.=5649, *P*=0.002) in the post-phase compared with the pre-phase. The discrepancy between results may be attributed to the difference in how stress levels were categorized. In the ordinal generalized mixed-effects model, response categories were collapsed into three categories from the original five levels due to smaller numbers of responses in the higher stress categories (stress levels 3 and 4). Thus, the ordinal generalized mixed-effects model could not detect the nuanced changes from one stress level to the next given the collapsed categories.

Our third hypothesis was that Black participants would report higher stress levels from the pre-phase to the during-phase compared with White participants. The ordinal generalized mixed-effect model showed no difference in the trajectory of stress from the pre-phase to the during-phase for Black vs. White participants (OR=1.2, 95% CI: 0.6, 2.5).  This finding was consistent with the results from the linear mixed-effects model, which showed that there was no difference between Black and White participants in the change in mean stress from the pre-phase to the during-phase (= -0.02, *P*=0.9).
